# Exploration of Learning during an International Health Elective Using Photovoice Methodology

**DOI:** 10.3390/pharmacy4040039

**Published:** 2016-11-29

**Authors:** Amy Werremeyer, Elizabeth Skoy, Gina Aalgaard Kelly

**Affiliations:** 1 School of Pharmacy, North Dakota State University, Fargo, ND 58108, USA; elizabeth.skoy@ndsu.edu; 2 Department of Sociology and Anthropology, North Dakota State University, Fargo, ND 58108, USA; gina.kelly@ndsu.edu

**Keywords:** photovoice, experiential learning, pharmacy, international health experience

## Abstract

Based on surveys and structured interviews, International Health Experiences (IHEs) improve cultural sensitivity, communication, and self-confidence among health professions students. However, open-ended methods to explore student learning during an IHE are not widely utilized. We sought to explore pharmacy student-identified learning during an IHE in an open-ended fashion using Photovoice methodology. Pharmacy students on an IHE in Guatemala were given disposable cameras and asked to photograph images that reflected their learning. Through the application of Photovoice methodology students captured, reflected upon, and presented photos to describe the learning they experienced. Themes were drawn from the reflective and focus group data collected. During three IHEs, six students captured seventy-seven photos. Four main learning themes emerged: culture/cultural competence, professional growth, shifting of attitudes, and meaningful/emotional experiences. Pharmacy students documented learning in expected (cultural competence, professional growth) and unexpected (emotional experiences) domains during an IHE. Photovoice may be an effective methodology for the exploration of learning, allowing students to capture their own learning including and beyond what is expected by their instructors.

## 1. Introduction

The higher education literature contains testimonials and reports summarizing the benefits and learning realized by health professions students during international health elective (IHE) training experiences throughout the health professions. The majority of this literature, incorporating quantitative methodology, such as structured interviews and survey instruments, has shown IHEs to be beneficial to students training in the healthcare professions through gains in cultural awareness and sensitivity, communication, self-efficacy, self-confidence, and compassion [1,2]. Qualitative analysis of student-generated IHE reports and open-ended survey questions have often agreed with findings from quantitative studies, but some have described occasional negative perceptions of IHEs as well as learning that occurred in unexpected domains [3,4,5]. In their evaluation of learning by medical students during an IHE, Holmes, et al found that open-ended questioning of students’ assessment of the value of their IHE revealed learning in unexpected areas, including self-reflection [5]. Holmes and colleagues suggest that students’ own learning objectives should be considered in evaluating learning during an IHE experience. When assessing learning in our newly developed IHE for pharmacy students, we sought an open-ended method in which students would be free to identify and describe learning that may take place within expected as well as unexpected domains.

Photovoice is a research method that lends itself to learning environments, both as a learning activity and a learning documentation tool. Pioneered by Wang and Burris, Photovoice is a process in which individual participants use cameras to photograph their everyday realities, thereby focusing on issues of greatest importance to them and communicating these issues to policy makers, administrators, healthcare providers, or others who can be mobilized to make change [6,7]. Its theoretical underpinnings, based on Freire’s approach to education for critical consciousness [8] and feminist theory, promote deep understanding of lived experiences and encourage the participant to construct and share his/her reality visually [9]. Photovoice has been used as an educational enhancement and research tool, in classroom and experiential learning settings [10,11,12]. Since Photovoice puts the “voice” in the hands of the participants, in educational settings it may be less likely to impose preconceived ideas of what a student should or would be learning as could be seen when administering structured survey instruments or interviews that have been utilized previously in the literature. Hence, the researchers chose Photovoice methodology for this exploratory study with the idea that it may give students the opportunity to describe their learning with few boundaries. Thus, the goal was to utilize Photovoice as a way to document and articulate student learning, seeking the students’ own perceptions of the direction of their learning. The intent of this article is to report the findings of this exploratory research using Photovoice methodology to identify student learning on an international medical mission-based IHE.

## 2. Materials and Methods

### 2.1. Setting

In the United States, student pharmacists are required to complete 200 h of experiential learning, entitled advanced pharmacy practice experiences (APPE), during their Doctor of Pharmacy (PharmD) education. These typically take place during the final year of a four-year professional pharmacy education program. The authors of this manuscript developed a new APPE, centered on an international medical mission to Guatemala [13]. This IHE, in total lasting 5 weeks, included ten days of travel throughout urban and rural areas in Guatemala, during which pharmacy students participated as part of a medical mission team. Prior to departure for Guatemala, pharmacy students spent 5 working days on the university campus preparing for their work abroad, engaging in self-directed study, and delivering four 30-min presentations regarding medications on the mission team formulary, tropical and non-tropical diseases and Guatemalan culture and language. The students were given a set of formal learning objectives associated with the IHE and were informed that they would be evaluated at the end of the 5 weeks based on the APPE evaluation rubric that is standard to the PharmD training program at our institution. At mission clinic sites during the 10 days of Guatemala travel, the pharmacy students operated a medication-dispensing hub, educated patients, and consulted with medical team members. The medical team ranged from 20 to 25 individuals which included physicians, advanced practice nurses, pharmacists, nurses, dentists, dental assistants and social workers. The pharmacy students’ activities were supervised by authors 1 and 3, who were faculty members fulfilling the role of pharmacy preceptors. Upon return from Guatemala to the United States, students spent 10 working days performing quality improvements, reporting paperwork, completing a written reflection, and preparing and delivering a final presentation. All of the above activities of the IHE were required for students, but with the exception of the written reflection, were not considered part of the research study.

The research was approved by the Institutional Review Board (IRB). Students opted to participate by giving their informed consent at the beginning of the APPE. Participation in the research was not a condition of nor did it affect their (pass/fail) APPE evaluation.

### 2.2. Participants

The Photovoice method in this study was applied to the sample of students selected for the IHE. The selection first involved interviews of pharmacy students who expressed interest in participating in the IHE, using non-validated interview questions. A total of nine students were interviewed over the three cohorts described here. The two pharmacy students in each interview cohort that displayed the greatest subjective qualities of flexibility, teamwork, and lack of self-centeredness based on interview responses were selected for the IHE as these qualities were most desired for medical mission team members. All students selected for the IHE were offered the opportunity to participate in the research. The sample size in each cohort, was limited to two pharmacy students due to size limitation of the medical team. All selected students were in their final (fourth) year of professional pharmacy education and their ages ranged from 23 to 27 years. Over 3 years, all six selected students (two students in each of three cohorts) opted to participate in the research. The resultant sample was representative of the larger pharmacy school demographics, including variety in age, gender, and prior culturally diverse experiences. Five of the students were female, and four of the students had previously traveled internationally, but none had traveled to Guatemala or had previously partaken in an IHE.

### 2.3. Procedures

Each student was given a 27-exposure disposable camera and was informed that he/she could take as many pictures as necessary, and did not have to use all 27 exposures. Students were asked to imagine that they were going to mount a photographic display with images that would showcase their learning to others. Students were instructed not to take photos in which individuals could be identified without the individual’s or their parent/guardian’s permission. According to the IRB approval, once permission was granted by an individual for their photo to be taken, participants and researchers were given the authority to publish captured photos. As an extra layer of protection, the authors have chosen to de-identify photos of individuals. Students were also instructed not to take photos of illegal or incriminating activity.

At the time of taking a photograph, students were asked to reflect on it by recording their thoughts in a journal that was distributed with the camera at the beginning of the study. Students were also asked to reflect in their journal daily during the 10-day trip. No formal reflection between the preceptors and the students nor between the students themselves was conducted while abroad. Informal discussions on learning between students and preceptors did occur, but this information was not collected. At the conclusion of the time in Guatemala, the cameras were collected from the students, and photos were developed in duplicate. The students were given a copy of their photos and journal entries.

**Figure 1:**
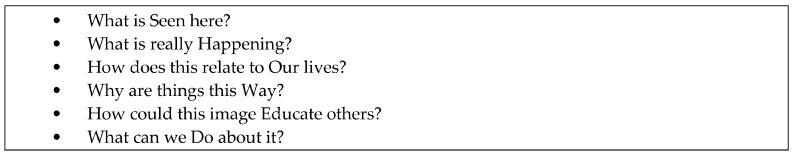
The SHOWED technique [14].

Two weeks after returning to the United States, student members of each cohort participated in a focus group led by Authors 1 and 2. Three separate focus groups were held, one for each cohort. During the focus groups, photos and journal entries were discussed and the SHOWED technique [14] (Figure 1) was used to facilitate discussion. Students elaborated upon their answers to the SHOWED questions, reflected on each other’s photos and responded to follow-up questions. The focus group sessions were transcribed by Author 1.

### 2.4. Analysis

Researchers collaborated to perform a thematic analysis of the data which consisted of photo-related journal entries, transcripts of focus groups, and formal written reflections. Computer software ATLAS.ti (ATLAS.ti ^TM^ GmbH, Berlin, Germany) was used to upload the data and allowed for an organized means of analyzing the data, but did not do any coding of concepts or themes. Therefore, codes needed to be created, organized and interconnected when appropriate by the researchers. Author 3 (a medical sociologist) conducted the first open and line-by-line coding, identifying initial emergent themes. Each time a theme was mentioned by a single participant, it was counted as a separate instance of that theme occurrence. Next, Authors 1 and 2 conducted further open and line-by-line coding to clarify coding and provide further structure and organization of emergent themes. At this juncture, all three researchers worked together to finalize theme structure and organize several sub-themes with similarities or common identifiers that were grouped together within the emergent themes. Consensus was used to handle discrepancies.

## 3. Results

In the three cohorts, all six students selected for the APPE also chose to participate in the research. All used cameras and journals to document and reflect upon their learning. A total of 77 photos were taken, and the number of photos taken per participant ranged from seven to 24. Four main themes emerged from the data and represented learning domains identified by the students. These were: attitude, professional growth, cultural competence shift, and in the field experiences (Table 1).

### 3.1. Attitude

**Figure 2:**
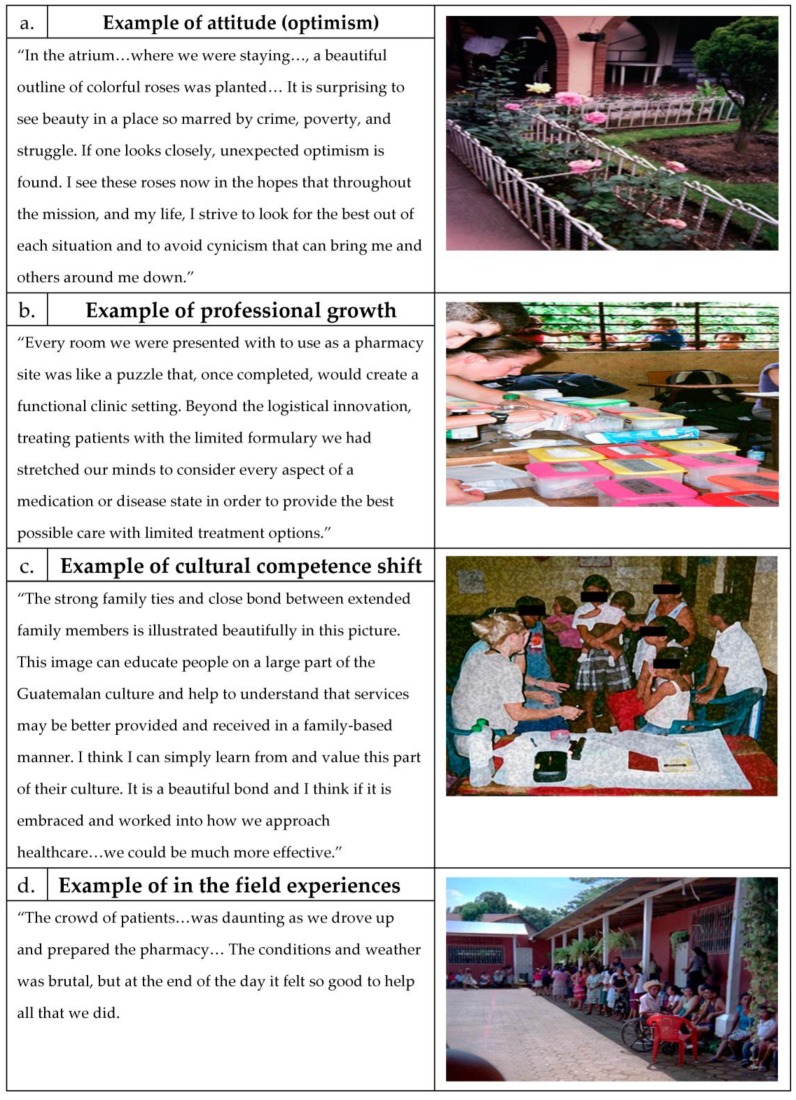
Examples of learning themes as captured by students on an international health experience.

This theme represents the students’ description and portrayal of a shifting of their attitudes during the APPE. This theme was identified by students’ use of words such as “took for granted”, “gratitude”, “optimism”, or “serving others”. In most cases, the students identified that their attitudes were changing in ways that were beneficial to them not only as medical professionals, but also as humans in general. The students also documented and reflected on times when they caught themselves correcting their own negative attitudes. The sub-themes of optimism, gratitude or serving others within this attitudinal theme typically surfaced in response to students’ experiences of working in conditions without the comforts or resources they were accustomed to. These experiences served to remind or instil in the students, at various times, appreciation for what they have (gratitude), a dedication to remain positive under adversity (optimism), or a commitment to help make things better for all people in the future (serving others). The images the students captured that served to portray their attitudinal changes were often of a mundane object for which they had new appreciation (Figure 2a) or one that was striking in its power to elicit empathy or the desire to serve on the part of the student.

### 3.2. Professional Growth

This theme represents the students’ description of the development and/or reinforcement of skills and abilities of a medical professional and confidence in those skills or abilities. This theme was identified by students’ use of words such as “expand clinical knowledge”, “confidence”, “working with other healthcare professionals”, “communication”, and “approach to patient education”. Each student captured at least one photo depicting the work of the interdisciplinary team providing patient care and commented on their realization of its value as well as confidence in operating within it. Each student also captured at least one photo of the pharmacy logistics (Figure 2b) and used it to portray their learning about one or more medical or logistical problem solved with limited resources or medication-specific knowledge gained. Finally, five of the six students captured an image reflecting that they had learned ways to improve their communication with patients, most of which could be applied to future patients as well.

### 3.3. Culture/Cultural Competence

This theme represents the students’ depiction of their recognition of cultural factors different from their own as well as changes or suggested changes in their own behavior (Figure 2c) in response to that recognition. This theme was identified by students’ use of words such as “traditions”, “culture”, and “respect for differences”. In their reflections and focus groups, all six students described how they began to recognize the significance of a differing lifestyle, food, poverty status, medical system and resource availability and how these factors feed into the values and norms of the Guatemalan people. They also described a growing recognition of how much they still needed to learn about this new culture in order to fully understand it. Three of the students described their growth in valuing the culture without feeling a need to alter it. Though most of the learning in this theme was described as positive, one student described becoming annoyed by cultural norms she observed, which led to a negative feeling about the people in general.

### 3.4. In the Field Experiences

This theme represents the students’ depiction of learning that became real because of the experience itself. It also encompassed emotions felt in response to those experiences that were often part of or intertwined with the learning itself. This theme was identified by students’ descriptions of their feelings as well as descriptions of places and people that, by their very presence, were instrumental in affecting the students’ learning (Figure 2d). Two students also described minor barriers to learning (occasional shortage of interpreters and food intolerance) during the APPE. All six students captured and described at least one emotional experience during the APPE. Emotions depicted by the students were generally positive and powerful, but not overwhelming. The student that captured the photo in Figure 2d used the word “daunting” to describe the emotional impact of seeing so much unmet healthcare need, but also the words “felt so good” to describe altruism and personal rewards of the experience. She added, “I think it’s hard (for others) to understand that feeling if you haven’t done this (before)”. Another student that captured a very similar photo to the one depicted in Figure 2d stated the following, “I saw this image everyday and it never got old, in fact, it only seeped deeper and deeper into my mind and heart”. Students also described emotions that were negative. For example, one student stated, “It irritated me that (another mission team member) didn’t care that some of the mistakes she was making could’ve been really dangerous”. The students universally summed up comments about negative emotions as being associated with a positive learning experience.

## 4. Discussion

We asked students to capture their learning visually using photographs. The themes that emerged from the analysis of their photos and reflections are illustrative of the domains within which the students identified learning taking place. The first three themes of learning captured by the students in this study are consistent with what has been reported in the literature previously [1,2] as well as with the objectives that were given to them prior to their travel. These are important learning domains for training of healthcare professionals and address requirements in Pharmacy educational standards [15,16,17]. Learning within these domains was expected and lends further support to the notion that IHEs are educationally beneficial.

The findings with regard to learning in the fourth theme, in the field experiences, as captured by our students, are not as frequently described in connection with learning during IHEs, especially the emotions aspect. The emphasis that the students placed on their emotions and the contribution of those emotions to learning during the trip was an unexpected theme. It is not that emotions themselves were unexpected. This IHE, and likely most IHEs around the world, placed students in challenging circumstances with high likelihood to induce emotions of many sorts. However, the emergence of emotions felt in the field as a major domain and contributor to student learning reveals a potentially important aspect of IHE learning that has not previously been explored. There has long been described an important and often complex interplay between learning and emotional experiences. Leading theories state that emotional experiences, especially positive ones, are powerful reinforcements for learning with emotions providing important contextual memory prompts [18,19]. The findings within the “in the field experiences” theme in this study are quite consistent with that notion. Our students very insightfully keyed in on the connection between their learning and emotional experiences when using Photovoice. This allowed them to reveal a learning theme that would not have been evident had the researchers supplied only their own pre-set learning objectives or domains. During a focus group reflection, one student stated “I didn’t remember having a rollercoaster of emotions when I was there…” indicating that even she had forgotten the degree to which her emotions affected her learning until returning to reflect on the visual reality of her experience that she had captured with her photos.

The students highlighting their emotions as an important contributor to their learning brings up several important areas that may need further attention with regard to IHEs. For example, should educational institutions that offer IHEs as part of their training programs prepare students for emotional experiences before they travel? How much more could emotional experiences be leveraged to enhance learning during an IHE where faculty and students prepared ahead of time to make connections between those emotions and learning? Should institutions perhaps consider requiring reflective or therapeutic sessions to help students deal with emotions during and after IHEs? These questions remain unexplored, to our knowledge. Our students described their emotional experiences as powerful, but not pathologic. Yet it is conceivable that some students could have difficulty coming to grips with emotions experienced during an IHE. An additional question that may also deserve further attention is whether a Photovoice approach to capturing student learning during an IHE is beneficial for identifying and revealing emotional aspects of learning as compared with other learning assessment methods. Furthermore, can application of Photovoice methodology to all types of experiential learning outside of IHEs encourage further connections between emotional experiences and learning? Findings describing the theme ‘love and happiness’ reported by Hernandez and colleagues [12] when employing Photovoice with college students engaging in a service learning activity suggest that this may be possible. Further application of Photovoice methodology and exploration of affective influences in the context of experiential learning may help to answer some of these questions.

Our findings should be viewed with some limitations in mind. First, the small size of the sample described (*n* = 6) makes it difficult to generalize the findings of the study. However, there is precedence for small sample sizes such as this according to previous published work utilizing Photovoice methodology [20,21,22]. In addition, the IHE described here may not be typical of all IHEs as instructional methods, pre-travel and post-travel training, geographical location, and cultural immersion may differ widely. In addition, it is possible that based on the interview selection criteria used, the students selected for this IHE may have been more likely to express or identify emotions than other students at the same level of training. Two of the authors conducting the research traveled with the students and assigned their overall grade for the IHE. This could have confounded the students’ responses and/or the faculty members’ interpretations of students’ reflective data. However, the data analysis by Author 3, who was not directly involved in travel or evaluation of student performance likely minimized potential bias of this nature. The use of Photovoice for the students’ description of their learning involved an average cost of twenty dollars per student, with the majority of that cost going toward photo development. The average time input by the faculty was one hour per student. Finally, though the photographic quality of a disposable camera was often not ideal, this was not an area of concern. The students commented that digital cameras would be more user-friendly and would produce better quality photos, but they recognized the value of the inability to alter their photos once they had taken them. They endorsed the notion that the photo captured in the moment of the experience was ideal, regardless of the photographic device or quality.

## 5. Conclusions

Through this research, students and faculty were able to identify learning that took place during a new IHE through the use of Photovoice. Learning was prominent in the areas of cultural competence growth, professional skill enhancement and changes in attitudes, which are expected and highly valuable learning domains. Additionally, students highlighted their emotional experiences in the field as a major contributor to their learning, which was an unexpected learning domain. Photovoice may represent a promising methodology for the exploration of learning during experiential training, allowing students to capture their own learning without the imposition of pre-conceived outcomes that may limit their learning identification. Future research incorporating Photovoice methodology in diverse experiential learning settings is necessary to confirm these findings.

## Figures and Tables

**Table 1:** Themes and sub-themes.

Theme	Attitude	Professional Growth	Culture/Cultural Competence	In the Field Experiences
Sub-themes	Optimism	Interprofessional team	Cultural traditions	Emotion
	Gratitude	Pharmacist skills	Medical systems	Barriers
	Humility	Knowledge gained	Recognition of cultural competence shift	Education versus experience
	Advocate for/pursue service experiences in the future	Communication skills		
		Innovation/flexibility		
